# Evaluation of Sexual Function Among Infertile Women and Their Sexual Self-Concept

**DOI:** 10.18502/jri.v21i4.4334

**Published:** 2020

**Authors:** Hedyeh Riazi, Hajar Lotfollahi, Reza Omani-Samani, Saman Maroufizadeh, Ali Montazeri

**Affiliations:** 1- Department of Midwifery and Reproductive Health, School of Nursing and Midwifery, Shahid Beheshti University of Medical Sciences, Tehran, Iran; 2- Students Research Office, School of Nursing and Midwifery, Shahid Beheshti University of Medical Sciences, Tehran, Iran; 3- Department of Epidemiology and Reproductive Health, Reproductive Epidemiology Research Center, Royan Institute for Reproductive Biomedicine, Tehran, Iran; 4- Department of Biostatistics, School of Nursing and Midwifery, Guilan University of Medical Sciences, Rasht, Iran; 5- Population Health Research Group, Health Metrics Research Center, Iranian Institute for Health Sciences Research, ACECR, Tehran, Iran

**Keywords:** Infertility, Sexual dysfunction, Sexual health, Sexual self-concept

## Abstract

**Background:**

The present study was designed to assess the association between sexual self-concept and sexual function in infertile women.

**Methods:**

A study with a convenience sample of women attending a referral infertility center (Royan Institute) was conducted in Tehran, Iran, in 2017. The Multidimensional Sexual Self-Concept Questionnaire (MSSCQ) and the Female Sexual Function Index (FSFI) were used to collect data. Chi-Square, t-test, Mann-Whitney U test and logistic regression were applied to analyze the data. The significance level was set at p<0.05.

**Results:**

The mean age of participants was 29.7±5.2 years. Overall, 152 women (60.8%) reported that they were experiencing sexual dysfunction. Comparing women with and without sexual dysfunction, there were significant differences between two groups on most measures such as sexual anxiety, sexual motivation, sexual satisfaction, and sexual depression (p<0.05). However, the results obtained from logistic regression indicated that women’s and husband’s age (OR for women’s age=1.26, 95% CI=1.10–1.44, p<0.001; OR for husband’s age=0.86, 95% CI=0.77–0.97, p=0.014), cause of infertility (OR for female factor=9.17, 95% CI=2.26–37.2, p=0.002; OR for male factor=3.90, 95% CI=1.26–12.1, p=0.018; OR for male and female factor=3.57, 95% CI=1.12–11.4, p=0.032), sexual motivation (OR=0.35, 95% CI=0.16–0.75, p= 0.007) and sexual satisfaction (OR=0.23, 95% CI=0.09–0.56, p=0.001) were significantly associated with sexual dysfunction.

**Conclusion:**

The findings suggest that sexual motivation and sexual satisfaction are important dimensions of sexual self-concept in infertile women. Indeed, it is essential to inform policy makers and stakeholders to provide more sexual health support for this population in the process of treatment.

## Introduction

Sexual function is an important aspect of quality of life that is linked to many known and unknown physical, psychological and emotional aspects such as sexual self-concept (1). Sexual self-concept is a complex issue and described as the emotions, opinions and beliefs that individuals have about their sexual relationships (2).

Studies have shown that sexual self-concept, though includes many constructs, could be measured and is very relevant to studies of sexual behaviors. Previous studies have shown that there is a significant relationship between sexual self-concept and sexual function in adolescents and women of reproductive age (3, 4). Indeed, sexual self-concept relates to biological, psychological and social factors (5), and at the same time, it could be influenced by other factors such as sexual self-efficacy and sexual self-esteem (5, 6).

It is argued that since sexual self-concept relates to one’s emotions, the concept might have stronger role among infertile women with altered emotions (7). However, little is known about sexual self-concept in infertile women. Thus, assessing sexual self-concept in infertile women is of prime importance since marital and sexual relationships among this population are usually reported to be sub-optimal (8). In fact, it is argued that lower level of sexual self-concept could lead to lower level of couples’ involvement, intimacy and sexual satisfaction (9, 10).

Some investigators pointed out social, emotional and mental disorders resulting from infertility in turn might lead to sexual matters and marital issues between couples (11). Meanwhile, what is most affected is sexual self-concept due to decreased motivations and excitements toward sexual issues and fade of romance in sexual life. Thus, when women’s feelings towards their gender role as a female are strongly influenced, then the sexual self-concept could be affected (7). The prevalence of infertility in Iran is reported about 20.2% that is noticeable (12). Given the importance of preserving and promoting the lives of infertile women, it seems necessary to conduct studies in this regard. Therefore, this study aimed to investigate the relationship between sexual self-concept and sexual function in infertile women. The findings from this study might help to design and implement appropriate interventions in order to improve sexual health in infertile women.

## Methods

### 

#### Participants and study design:

A study with a convenience sample of women attending a referral infertility center (Royan Institute) was conducted in Tehran, Iran, in 2017. The sample size was calculated based on estimation of prevalence of sexual dysfunction among infertile women (80%) (13). Therefore, the required sample size for the study with 80% power and d=0.05 (Precision 5%) at 5% significance level was estimated to be 246 women. However, in practice, 250 women were included in the study.

The inclusion criteria were as follows: being infertile for at least one year, being 18 to 45 years old and having the ability to read and write in Persian. Participants were asked to complete the study questionnaires while waiting to visit doctors.

### Questionnaires:

#### The Multidimensional Sexual Self-Concept Questionnaire (MSSCQ):

It is a 100-item self-report instrument measuring the various dimensions of sexual self-concept. It was developed by Snell in 1990 to assess the following 20 domains: sexual anxiety, sexual self-efficacy, sexual consciousness, motivation to avoid risky sex, chance/luck sexual control, sexual preoccupation, sexual assertiveness, sexual optimism, sexual problem self-blame, sexual monitoring, sexual motivation, sexual problem management, sexual esteem, sexual satisfaction, power-other sexual control, sexual self-schemata, fear of sex, sexual problem prevention, sexual depression, and internal sexual control (14). Each domain contains 5 items and is rated on a five-point Likert scale ranging from 0 (Not at all characteristic of me) to 4 (Very characteristic of me). The average score for each domain is considered as the cut-off point and a score above the average indicates the higher desire in that domain. The psychometric properties of the Persian version of MSSCQ are well documented (15).

#### The Female Sexual Function Index (FSFI):

It is a 19-item self-administered questionnaire designed to assess female sexual function. It contains six domains: desire (2 items), arousal (4 items), lubrication (4 items), orgasm (3 items), satisfaction (3 items), and pain (3 items). Questions are scored based on a Likert scale and total score of FSFI could be obtained by summing up the scores achieved in each domain. The FSFI total score ranges from 2 to 36 with higher scores indicating better sexual function (16). The Iranian version of FSFI indicates that it is a reliable and valid instrument for measuring sexual function in women where a score of 28 is considered as cut-off value for Iranian population (17).

### Statistical analysis:

Data were analyzed using SPSS version 16. Descriptive analysis was performed to explore the data. Chi-square, t-test and Mann-Whitney U test were applied to analyze the data. The significance level was set at p<0.05. Logistic regression analysis was used to assess the relationship between sexual function (Dependent variable) and sexual self-concept while adjusting for demographic and reproductive factors (Independent variables). Accordingly, using the “enter method”, adjusted odds ratio (OR) with 95% confidence interval (CI) was reported. Demographic variables included recording of women’s and husbands age, educational level, employment and economic status. The information on economic status was self-reported with three response categories of good, intermediate and poor.

#### Ethical consideration:

The research code of ethics was obtained from the Ethics Committee of Shahid Beheshti University of Medical Sciences (IR. SBMU.PHNM.1394.263). For sampling, the researcher first introduced herself to the candidates, explained the study objectives and ensured them of their anonymity and the confidentiality of their data, and distributed the questionnaires among them after they had completed the written consent forms.

## Results

A total of 250 infertile women participated in the study. The mean age of participants was 29.7±5.2 years. The majority were unemployed (78.8%), and had intermediate economic status (71.6%). The characteristics of study participants are shown in table 1. The mean score for sexual function was 25.4 (SD=6.1) and 60.8% of women reported sexual dysfunction.

**Table 1.** The characteristics of the study samples (n=250)

	**All (n=250)**	**FSFI ≥28 (n=98)**	**FSFI <28 (n=152)**	p^*^
**Women’s age (Years) (Mean, SD)**	29.7 (5.2)	29.2 (5.0)	30.1 (5.4)	0.199
**Husband’s age (Years) (Mean, SD)**	33.9 (5.2)	34.1 (4.9)	33.8 (5.5)	0.639
**Education (No.,%)**				0.092
Primary	37 (14.8)	9 (9.2)	28 (18.4)	
Secondary	91 (36.4)	35 (35.7)	56 (36.8)	
Higher	122 (48.8)	54 (55.1)	68 (44.7)	
**Employment (No.,%)**				0.206
Housewife	197 (78.8)	73 (74.5)	124 (81.6)	
Employed	53 (21.2)	25 (25.5)	28 (18.4)	
**Economic status (No.,%)**				0.068
Poor	39 (15.6)	9 (9.2)	30 (19.7)	
Intermediate	179 (71.6)	74 (75.5)	105 (69.1)	
Good	32 (12.8)	15 (15.3)	17 (11.2)	
**Duration of marriage (Years) (Mean, SD)**	7.1 (3.9)	7.1 (4.0)	7.1 (3.8)	0.904
**Duration of infertility (Years) (Mean, SD)**	5.5 (3.8)	5.3 (3.7)	5.6 (3.9)	0.541
**Cause of infertility (No.,%)**				0.186
Female factors	54 (21.6)	18 (18.4)	36 (23.7)	
Male factors	87 (34.8)	34 (34.7)	53 (34.9)	
Male and female factors	68 (27.2)	24 (24.5)	44 (28.9)	
Unknown	41 (16.4)	22 (22.4)	19 (12.5)	
**Intercourse/month (Mean, SD)**	8.5 (4.5)	9.7 (4.6)	7.8 (4.2)	0.001

SD: Standard deviation.

* Derived from Chi-square or t-test

The comparison of sexual self-concept subscales between women with and without sexual dysfunction is presented in table 2. The results showed that 14 out of 20 subscales were significantly different between these two groups.

**Table 2.** The association between MSSCQ subscales and FSFI (n=250)

	**FSFI ≥28 (n=98)**	**FSFI <28 (n=152)**	

	**Median (IQR)**	**Median (IQR)**	p ^*^
**Sexual anxiety**	0.40 (0.80)	0.90 (1.00)	<0.001
**Sexual self efficacy**	2.80 (0.80)	2.20 (0.80)	<0.001
**Sexual consciousness**	2.90 (0.60)	2.40 (0.80)	<0.001
**Motivation to avoid risky sex**	3.40 (0.80)	3.00 (1.00)	0.016
**Chance/luck sexual control**	0.60 (0.80)	0.80 (1.00)	0.012
**Sexual preoccupation**	0.60 (1.00)	0.80 (1.00)	0.399
**Sexual assertiveness**	1.90 (0.80)	1.80 (0.60)	0.001
**Sexual optimism**	1.80 (0.60)	1.80 (0.80)	0.411
**Sexual problem self blame**	1.40 (0.85)	1.60 (1.00)	0.240
**Sexual monitoring**	1.00 (0.80)	1.00 (0.95)	0.111
**Sexual motivation**	2.60 (0.80)	2.20 (0.80)	<0.0001
**Sexual problem management**	2.60 (0.65)	2.30 (0.80)	0.001
**Sexual esteem**	3.00 (1.00)	2.40 (1.00)	<0.0001
**Sexual satisfaction**	3.20 (0.80)	2.20 (1.00)	<0.0001
**Power other sexual control**	0.60 (0.80)	0.80 (0.80)	0.118
**Sexual self schemata**	3.80 (0.60)	3.40 (1.00)	<0.0001
**Fear of sex**	1.60 (1.05)	1.60 (1.00)	0.225
**Sexual problem prevention**	3.20 (0.65)	2.80 (1.20)	<0.0001
**Sexual depression**	0.00 (0.40)	0.60 (1.00)	<0.0001
**Internal sexual control**	2.60 (0.80)	2.40 (0.80)	0.001

IQR: Interquartile Range.

* Derived from Mann-Whitney U test

The results of regression analysis indicated that there was significant association between sexual dysfunction and women’s age (OR=1.26, 95% CI=1.10–1.44, p<0.001), husbands’ age (OR= 0.86, 95% CI=0.77–0.97, p=0.014), female factors as the cause of infertility (OR=9.17, 95% CI= 2.26–37.2, p=0.002), male factors as the cause of infertility (OR=3.90, 95% CI=1.26–12.1, p=−0.018), male and female factors (OR=3.57, 95% CI=1.12–11.4, p=0.032), sexual-motivation (OR=0.35, 95% CI=0.16–0.75, p=0.007) and sexual satisfaction (OR=0.23, 95% CI=0.09–0.56, p=0.001). However, there was no significant association between sexual dysfunction and other independent variables studied. The detailed results are shown in table 3.

**Table 3.** The results obtained from logistic regression analysis indicating odds ratio for sexual dysfunction among infertile women (n=250)

	OR (95% CI) ^*^	**p**
**Women’s age**	1.26 (1.10–1.44)	<0.001
**Husbands’ age**	0.86 (0.77–0.97)	0.014
**Education**		
Higher	1.0 (ref)	
Secondary	1.14 (0.42–3.07)	0.78
Primary	4.19 (0.98–17.8)	0.052
**Employment**		
Employed	1.0 (ref)	
Housewife	1.31 (0.44–3.92)	0.62
**Income**		
Good	1.0 (ref)	
Intermediate	0.79 (0.23–2.69)	0.71
Poor	3.89 (0.70–21.5)	0.11
**Duration of marriage**	0.90 (0.72–1.12)	0.35
**Duration of infertility**	0.98 (0.79–1.23)	0.91
**Cause of infertility**		
Unexplained	1.0 (ref.)	
Female factors	9.17 (2.26–37.2)	0.002
Male factors	3.90 (1.26–12.1)	0.018
Male and female factors	3.57 (1.12–11.4)	0.032
**Intercourse frequency per month**	0.93 (0.83–1.03)	0.18
**Sexual anxiety**	0.70 (0.26–1.84)	0.47
**Sexual self efficacy**	1.08 (0.37–3.11)	0.84
**Sexual consciousness**	0.50 (0.17–1.44)	0.20
**Motivation to avoid risky sex**	1.52 (0.69–3.35)	0.29
**Chance/luck sexual control**	1.39 (0.66–2.91)	0.37
**Sexual preoccupation**	1.16 (0.58–2.32)	0.66
**Sexual assertiveness**	2.23 (0.81–6.14)	0.12
**Sexual optimism**	1.25 (0.52–2.99)	0.60
**Sexual problem self blame**	0.77 (0.42–1.40)	0.39
**Sexual monitoring**	1.24 (0.58–2.64)	0.56
**Sexual motivation**	0.35 (0.16–0.75)	0.007
**Sexual problem management**	1.39 (0.56–3.49)	0.48
**Sexual esteem**	0.68 (0.26–1.80)	0.44
**Sexual satisfaction**	0.23 (0.09–0.56)	0.001
**Power other sexual control**	1.56 (0.77–3.17)	0.21
**Sexual self schemata**	2.19 (0.87–5.52)	0.09
**Fear of sex**	1.24 (0.60–2.56)	0.55
**Sexual problem prevention**	0.45 (0.18–1.09)	0.07
**Sexual depression**	2.37 (0.79–7.01)	0.12
**Internal sexual control**	1.01 (0.43–2.34)	0.98

* Adjusted for women’s age, husbands’ age, education, employment, income, duration of marriage, duration of infertility, cause of infertility, intercourse frequency per month and sexual self-concept using the “enter method”

## Discussion

The findings from this study showed that there was significant association between some aspects of sexual self-concept and sexual function in infertile women. The findings proved that sexual motivation and sexual satisfaction were the most important components of sexual self-concept that contributed to sexual function in infertile women. Sexual satisfaction and sexual motivation reduce sexual dysfunction by 0.77 and 0.65, respectively. In addition, the study findings indicated that sexual function in infertile women was associated with couples’ age and the cause of infertility as expected.

Sexual motivation is an important factor for initiating sexual behaviors (18). The results showed that female sexual function was affected by sexual motivation. This finding is consistent with other studies (19–21). It is argued that there are so many reasons for sexual motivation (22). It is possible to consider stress reduction, self-esteem boosting and goal attainment as common reasons; for example, having a child can be a sexual motivation factor in infertile women. Perhaps improving these motivating factors and directing them into pleasure, love and commitment results in further promotion of infertile women’s sexual function.

Sexual function was positively affected by sexual satisfaction. Sexual satisfaction brings positive emotions, beliefs and impressions about sexual communication and in general positive attitude towards sexual function. Hence, it is predictable that lower sexual satisfaction can be associated with sexual dysfunctions. This is confirmed in different studies (23, 24). Negative thoughts during sexual activity such as worrying about failure in fertilization and its unfortunate consequences are associated with sexual dissatisfaction (25). So, it is valuable to eliminate such intervening factors that reduce sexual satisfaction in infertile women.

The association between sexual dysfunction and age was evident where the probability for sexual dysfunction was higher in older women. In this regard, it seems that there is no difference between infertile women compared with fertile population. This is in line with findings from other studies (26–29). Since the analysis reports on sexual dysfunction in women, thus as indicated, 1-year increase in women’s age could increase the sexual dysfunction by 26%, while at the same time, 1-year increase in women’s age, could decrease sexual dysfunction in women’s husbands by 14% (Table 3). Perhaps one might argue this could be happening due to a better experience in sexual behavior during the process of intercourse by male partners. On the other hand, with increasing age and failing to achieve the main goal of sex which is pregnancy in an infertile woman, her attitude toward sexual issues changes and may lead to sexual dysfunction.

The results of our study demonstrated that the cause of infertility was an important affecting factor of sexual function. The odds ratio for sexual dysfunction in infertile women due to female factors was nearly three times more than other infertility related factors. This finding confirms that in this situation sexual relationship becomes an action of child producing rather than focusing on the pleasure. Therefore, one might argue that for women who suffer from female infertility, sexual dysfunction is not unexpected. This finding is in line with a Turkish study (26) perhaps due to the proximity of the culture of Iran and Turkey, although Kucur Suna et al. did not find any association between the causes of infertility and sexual dysfunction (30). Asking for separation from the spouse and remarriage is another problematic issue in the case of infertility due to female factors. One study from Cyprus showed that infertility was considered as a simple problem that can be easily treated with the advancement of technology and cannot be a reason for separation (31). The cultural context seems to play an important role in this regard. However, these outcomes and consequences can have disturbing effects on sexual function.

### Strengths and limitations:

The most notable strength of this study was the fact that this small piece of work for the first time reports and documents the association between sexual self-concept and sexual function among infertile women in Iran. Providing even descriptive evidence might help potential investigators to develop the topic further. However, this study had some limitations. First, the study was cross sectional in nature and used a convenience sampling method. Accordingly, the results should not be generalized to all infertile women and secondly they should be interpreted with caution. A better design is recommended for the future investigations. Additionally, it would be interesting to include infertile women with different backgrounds to understand if there would be differences among different subgroups of infertile women with regard to sexual self-concept and sexual satisfaction.

## Conclusion

The present study showed that sexual motivation and sexual satisfaction play an important role in sexual function among infertile women. Perhaps improving these dimensions of sexual self-concept might enhance sexual function among this population. Indeed, it is essential to inform policy makers and stakeholders to provide more sexual health support for this population in the process of treatment.
